# Persistent Epsilon-Like Wave After Right Atrial Myxoma Resection: A Case Report

**DOI:** 10.7759/cureus.96381

**Published:** 2025-11-08

**Authors:** Adrian A Naoun, Génesis Virella, Carol N Sánchez Santana, Milianette Cruz, Luis Jiménez Reyes

**Affiliations:** 1 Medicine, San Juan Bautista School of Medicine, Caguas, USA; 2 Electrophysiology, Caribbean Heart Institute, Caguas, USA

**Keywords:** echocardiography, ekg abnormalities, electrophysiology, epsilon wave, myxoma

## Abstract

Epsilon waves are a major electrocardiographic criterion of arrhythmogenic right ventricular cardiomyopathy (ARVC), but have also been described in inflammatory and infiltrative cardiomyopathies. Postoperative arrhythmias, most commonly atrial fibrillation, are well recognized following atrial myxoma resection; however, persistent epsilon-like activity in the absence of residual disease has not been previously documented. We report the case of a 46-year-old woman presenting with exertional dyspnea and vertigo who was found to harbor a large pedunculated right atrial myxoma on transthoracic echocardiography. Surgical excision yielded a 35-gram tumor, with histopathology revealing chronic inflammatory infiltrates and hemorrhagic foci. Postoperative imaging confirmed normalization of right-sided pressures with no residual mass, correlating with symptomatic resolution. Nevertheless, follow-up electrocardiography at eight months demonstrated a persistent epsilon-like deflection in lead V1, accompanied by right-sided conduction delay and T-wave inversion, while other nonspecific abnormalities resolved. The persistent ARVC-mimicking phenotype likely reflects localized fibrotic and inflammatory conduction system remodeling, aligning with mechanistic data that inflammation and matrix remodeling can delay right ventricular activation. This rare electrophysiological sequela of right atrial myxoma carries notable implications for rhythm surveillance and long-term risk stratification. Furthermore, future research should explore the role of immunophenotyping and advanced imaging in predicting postoperative recovery and facilitating targeted therapeutic strategies.

## Introduction

Primary cardiac tumors are rare, with an autopsy incidence ranging from 0.001 to 0.3% [1], and myxomas constitute the most common subtype. Tumorigenesis is epidemiologically governed by a sporadic or familial pattern exhibiting a 3:1 female-to-male ratio [2]. Cardiac myxomas predominantly originate in the left atrium (75%) and, less frequently, in the right atrium (15-20%) [3,4]. The histogenesis of cardiac myxomas is incompletely understood, yet multipotent mesenchymal stem cells are paradigmatically postulated as primitive precursors [5]. Although biologically benign, myxomas inherently carry an embolization tendency associated with complications such as stroke, transient ischemic attack (TIA), and neurological deficits [6].

The cornerstone of cardiac myxoma diagnosis encompasses echocardiography, as a spectrum of poorly specific clinical features may obfuscate diagnosis. Cardiac myxomas may manifest asymptomatically or exhibit a clinical trajectory comprising a triad of intracardiac obstruction, embolic events, and nonspecific constitutional manifestations mimicking alternative diagnoses [7,8]. Echocardiography is the gold standard for screening, followed by cardiac magnetic resonance and computed tomography for establishing the diagnosis [9]. Treatment comprises minimally invasive surgery with histopathological characterization of the mass; however, a timely diagnosis is vital to elude fatal complications [10]. Postoperative rhythm disturbances, most commonly transient atrial fibrillation, occur in roughly one-quarter of patients after resection [11]. In accordance with Heart Rhythm Society and American Heart Association guidance, continuous arrhythmia monitoring for 48-72 hours postoperatively and periodic ECG or Holter evaluation during the first year are recommended to confirm rhythm stability and detect late sequelae [12].

## Case presentation

**Figure 1:**
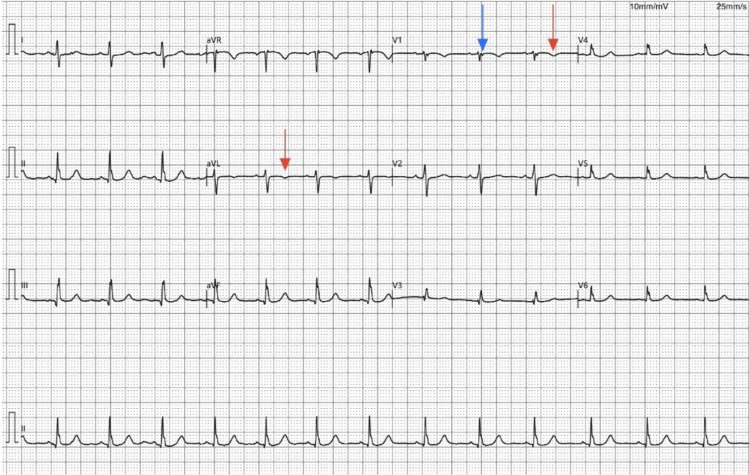
Initial electrocardiographic (EKG) evaluation. The EKG revealed inverted T waves in leads aVL and V1 (red arrow), poor R-wave progression (PRWP), nonspecific ST-T wave changes (NSSTTWC), and a right ventricular conduction delay (RVCD) with a primitive epsilon wave in lead V1 (blue arrow).

**Figure 2:**
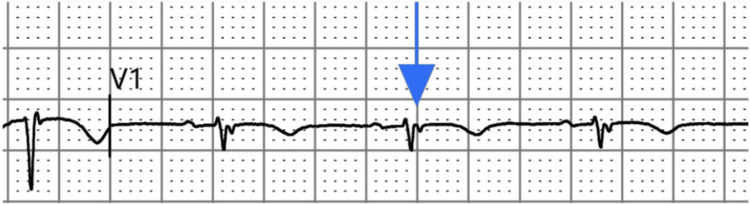
V1 lead. Magnified lead V1 demonstrates the primitive epsilon-wave notch (blue arrow) corresponding to delayed right ventricular depolarization.

Herein, a 46-year-old female presented with intermittent palpitations, accompanied by fatigue, vertigo, exertional dyspnea, and intermittent fevers over six months. Medical history was notable for former tobacco use, hypertension, hyperlipidemia, and hypothyroidism, treated with losartan (25 mg), simvastatin (20 mg), and levothyroxine (0.125 mg) QD, respectively. Family history included maternal hypertension and paternal coronary artery bypass grafting. On presentation, vital signs included a blood pressure of 136/95 mmHg, a heart rate of 86 beats per minute, a respiratory rate of 17 breaths per minute, and a body mass index (BMI) of 31.4. The physical examination was unremarkable, with no murmurs, pedal edema, or jugular venous distension. Electrocardiogram (EKG) evaluation revealed sinus rhythm with a heart rate of 82 bpm, P wave duration of 94 milliseconds (ms), PR interval of 138 ms, QRS duration of 78 ms, and QT/QTc interval of 361/423 ms, all within normal limits (Figures 1, 2). Baseline Holter monitoring revealed no arrhythmias, confirming sinus rhythm stability despite electrocardiographic abnormalities.

**Figure 3:**
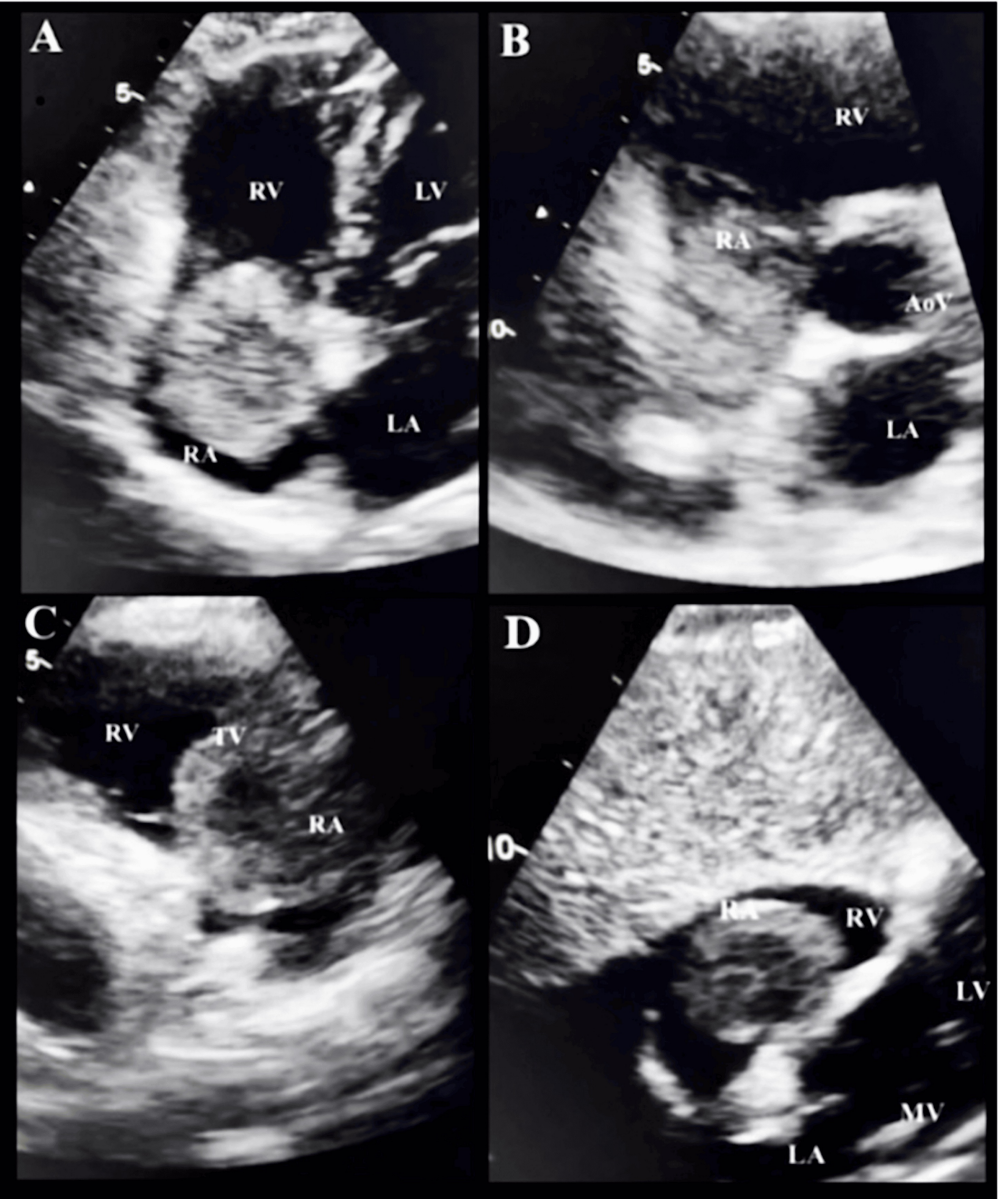
Two-dimensional transthoracic echocardiogram (TTE). (A) Apical view of four chambers, (B) short-axis view, (C) parasternal right atrial view, (D) subcostal view.

Given these inconclusive findings, a 2D transthoracic echocardiogram (TTE) was obtained, revealing a left ventricular ejection fraction of 65-70%, impaired diastolic relaxation, mild left atrial dilation, and a large pedunculated mass measuring 4.7 × 4.5 × 3.5 cm, consistent with a right atrial myxoma (Figure 3). The patient was initiated on warfarin to reduce embolic risk and referred for cardiothoracic surgery, with follow-up evaluation performed one month and 25 days after the initial consult. Transesophageal echocardiography (TEE) was not performed, as TTE provided adequate delineation of the tumor's septal attachment and hemodynamic impact. Preoperative coronary angiography was likewise not indicated, consistent with her estimated functional capacity ≥ 4 METs (metabolic equivalents) and preserved ventricular function, in accordance with the current American College of Cardiology (ACC)/American Heart Association (AHA) (2024) and European Society of Cardiology​​​​​​​ (ESC) (2022) guidelines, favoring selective evaluation.

**Figure 4:**
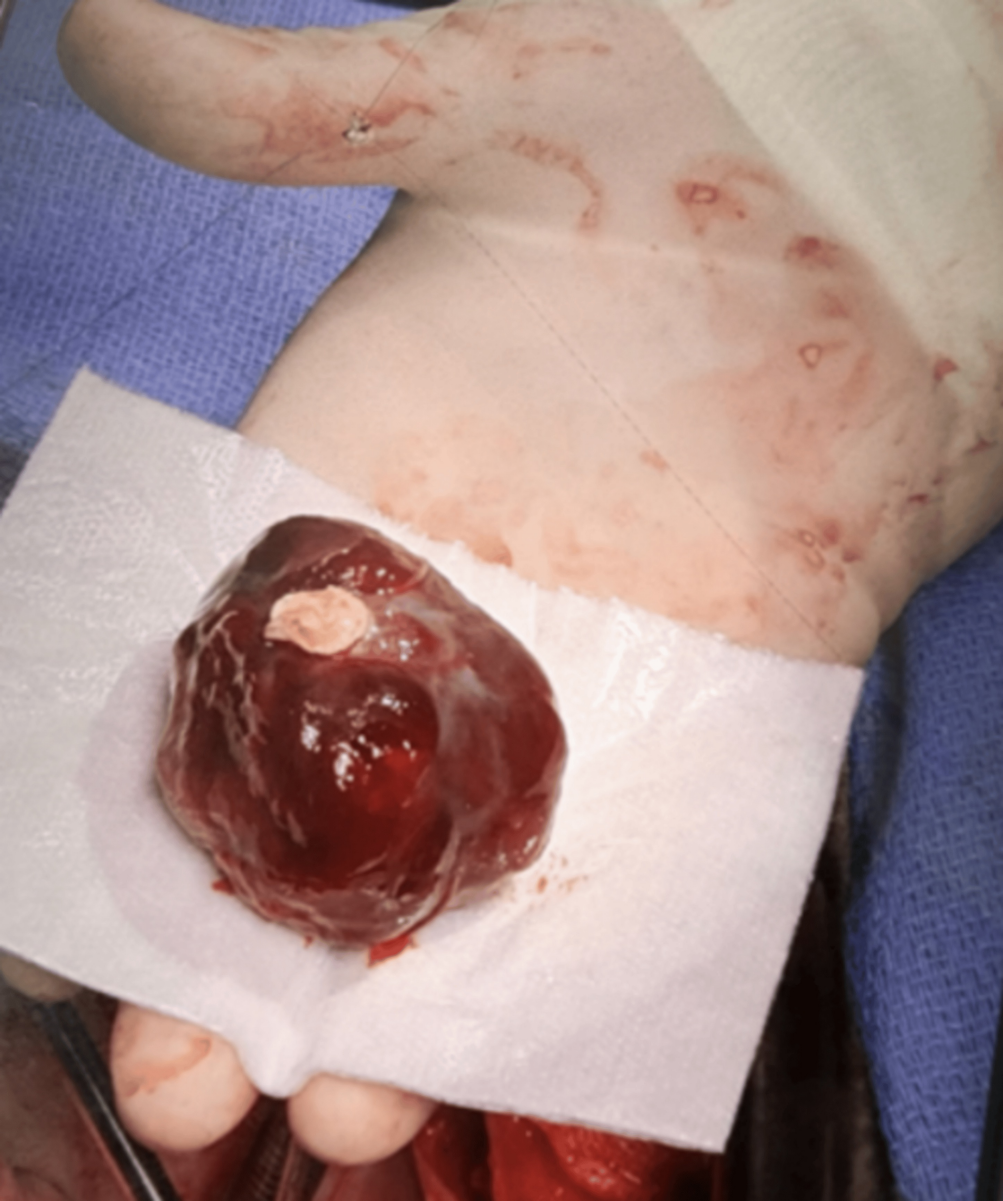
Gross surgical specimen. A smooth, glistening, and red-purple mass was excised two months and three days after the initial consult.

Upon admission, the patient reported experiencing chest pain, vertigo, and intermittent fatigue; however, the physical examination remained unremarkable. The surgical intervention involved performing a right atriotomy with excision of the septum-affixed mass and closure with 4-0 Prolene monofilament sutures. The 35-gram specimen measured 4.7 × 4.5 × 3.5 cm, accompanied by a 1.3 × 1 × 0.2 cm stalk (Figure 4). The histopathological examination confirmed the diagnosis of cardiac myxoma and revealed multiple dark red hemorrhagic foci with a chronic inflammatory infiltrate, suggesting a proinflammatory phenotype with potential postoperative sequelae.

**Figure 5:**
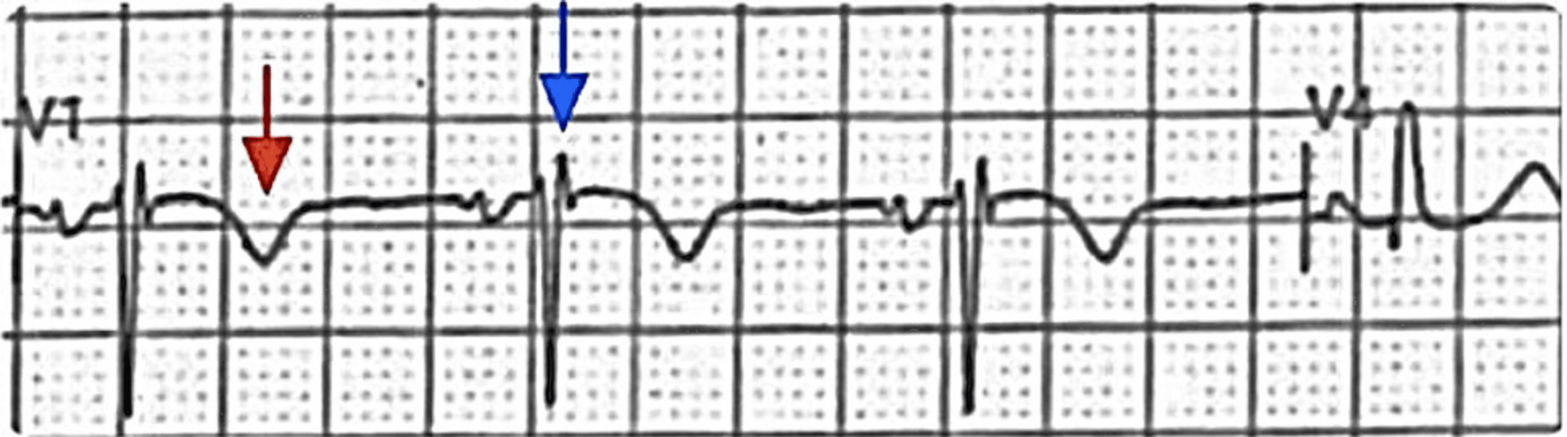
Magnified V1 lead (post-surgical). Zoomed lead V1 shows the epsilon-wave notch (blue arrow) and persistent negative T-wave deflection (red arrow), indicating localized conduction remodeling.

Despite the successful resection, follow-up EKGs at eight months demonstrated persistent inverted T waves in leads aVL and V1, an epsilon-like deflection in V1 associated with right ventricular conduction delay (RVCD), and right axis deviation, although nonspecific ST-T wave changes (NSSTTWC) and poor R-wave progression (PRWP) had resolved (Figure 5). Follow-up TTE excluded structural abnormalities, residual mass, or right atrial appendage thrombus and demonstrated physiological inspiratory collapse of the inferior vena cava (IVC), consistent with a right atrial pressure of 3 mmHg (improved from preoperative 8-10 mmHg). Repeat Holter monitoring and 72-hour postoperative telemetry revealed no arrhythmias, with preservation of right ventricular function. These persistent electrophysiological abnormalities suggest localized conduction remodeling, representing a rare arrhythmogenic right ventricular cardiomyopathy (ARVC)-like phenotype persisting after myxoma resection.

## Discussion

Primary cardiac tumors are rare, occurring at a frequency 20-40 times lower than secondary or metastatic cardiac neoplasms, most commonly from pulmonary (36-39%) and hematologic malignancies (12-21%), followed by breast cancer (10-12%) [13]. In contrast, approximately 75% of primary cardiac tumors are benign, with cardiac myxomas comprising half of these cases and representing the most common primary cardiac neoplasm, whereas the remaining 25% are malignant [14]. Cardiac myxomas demonstrate a heterogeneous clinical course, ranging from asymptomatic disease to presentations dominated by intracardiac obstruction, embolic phenomena, or constitutional symptoms, with variability influenced by tumor size, mobility, and location (Table 1) [14].

**Table 1:** Clinical manifestations of right and left atrial myxomas. Clinical manifestations are dictated by multiple variables, including tumor size, mobility, location, physical activity, and hemodynamic positioning. Table Credits: Created by the authors using original data synthesis; not reproduced or adapted from any copyrighted source.

Complications	Right Atrial Myxoma (RAM)	Left Atrial Myxoma (LAM)
Intracardiac obstruction	Right-sided heart failure: jugular venous distension, congestive hepatomegaly, peripheral edema, reduced right ventricular filling due to tricuspid valve obstruction, and possible acute pulmonary hypertension	Left-sided heart failure: pulmonary edema, dyspnea, orthopnea, and reduced left ventricular filling due to mitral valve obstruction
Embolization	Risk of embolism to peripheral organs, and right atrial thrombus formation resulting in pulmonary embolism	The risk of pulmonary embolism is rare, but can lead to cerebral stroke due to left atrial thrombus formation
Constitutional symptoms	Fever, fatigue, and weight loss	Fever, fatigue, and weight loss
Other symptoms	Syncopal episodes	Early or mid-diastolic murmur (mitral stenosis), and syncopal episodes

We herein describe a rare case of a right atrial myxoma complicated by persistent post-resection electrophysiological abnormalities, notably an epsilon-like wave, despite complete excision and normalization of right atrial pressures. Epsilon waves reflect delayed right ventricular depolarization and are classically associated with ARVC, where fibrofatty infiltration disrupts conduction [15]. Similar patterns, however, have been reported in cardiac sarcoidosis, myocarditis, Chagas disease, and post-infarction scar cardiomyopathy, conditions unified by inflammation-induced fibrosis [16]. To our knowledge, persistent postoperative epsilon-like activity after right atrial myxoma resection without arrhythmia has not been previously reported.

In our case, continuous postoperative telemetry and repeat Holter monitoring confirmed the absence of arrhythmias, and right-ventricular systolic function remained preserved on serial imaging. The persistent epsilon-like activity likely reflects localized conduction delay secondary to fibrosis within an inflammatory and hemorrhagic microenvironment, as supported by histopathology. The absence of arrhythmogenesis or ventricular dysfunction indicates a benign electrophysiologic course, provided routine rhythm surveillance is maintained. This inflammatory milieu has been associated with overexpression of cytokines such as interleukin-6 (IL-6) and tumor necrosis factor-alpha (TNF-α), which stimulate fibroblast activation, extracellular matrix (ECM) deposition, and progressive myocardial fibrotic remodeling [17]. Additional mediators, including vascular endothelial growth factor (VEGF), platelet-derived growth factor (PDGF), and monocyte chemoattractant protein-1 (MCP-1), further amplify stromal remodeling and immune cell recruitment. In addition, matrix metalloproteinases (MMP-2, MMP-9, and MT1-MMP) have been identified in papillary myxomas, contributing to ECM degradation, tumor fragility, and inflammation-driven remodeling [18].

**Figure 6:**
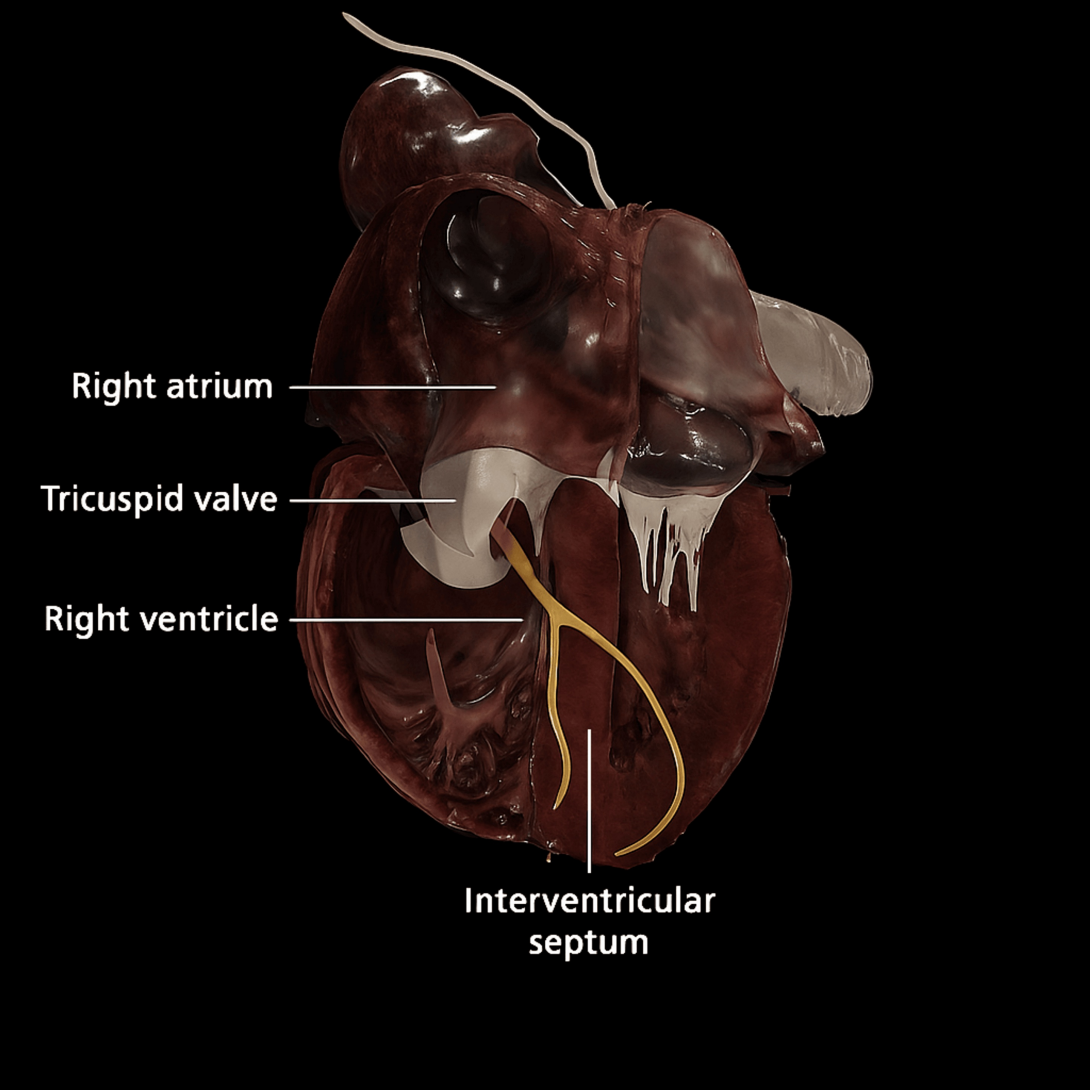
Three-dimensional anatomical illustration of the right atrium and adjacent structures. The image depicts the right atrium (RA), tricuspid valve, right ventricle (RV), and interventricular septum (IVS) to demonstrate the proximity between myxomatous lesions and conduction pathways. Chronic inflammation and fibrosis in this region may underlie delayed depolarization patterns such as the postoperative epsilon-like wave. Image Credits: Original 3D reconstruction generated using the Anatomage® digital anatomy platform (Anatomage Inc., Santa Clara, CA, USA) with institutional approval. Permission for reproduction was obtained and disclosed in the Acknowledgements.

Recent single-cell profiling of cardiac myxomas has confirmed that these tumors harbor an immunosuppressive and pro-fibrotic microenvironment. Jiang et al. (2024) demonstrated that myxoma cells exhibit a fibroblast-like transcriptional program, while tumor-associated macrophages expressing growth-promoting and angiogenic mediators (e.g., PDGFC, EREG) interact with fibroblasts enriched in extracellular matrix-modifying genes (e.g., *POSTN*, *COL1A* family), collectively supporting stromal remodeling, fibrosis, and T-cell dysfunction [19]. In parallel, the broader oncologic literature describes the role of cancer-associated fibroblasts contributing to immune evasion through cytokine release and checkpoint signaling, perpetuating chronic inflammation and impaired cytotoxic T-cell activity [20]. Together, these findings support the existence of a disease-specific immunosuppressive niche that may extend into adjacent myocardium, providing a plausible mechanism for localized conduction remodeling and the persistence of epsilon-like activity even after complete excision (Figure 6).

The right atrium (RA), tricuspid valve, right ventricle (RV), and interventricular septum (IVS) are labeled to demonstrate the proximity of myxomatous lesions to conduction system structures. The conduction pathways traversing the septum and adjacent myocardial tissue may be vulnerable to compression or inflammatory states. Chronic inflammation, accompanied by fibrosis and local remodeling, may contribute to persistently delayed depolarization patterns such as the epsilon-like wave observed post-resection. Although cardiac MRI offers superior spatial resolution for detecting myocardial fibrosis, inflammation, or subtle right-ventricular anomalies, confirmatory testing was not financially feasible for the patient. Serial echocardiography nevertheless confirmed complete structural and hemodynamic normalization, supporting the interpretation that the persistent epsilon-like activity reflected localized conduction remodeling rather than residual or recurrent pathology.

## Conclusions

Our case suggests that chronic inflammation and its electrophysiological sequelae may persist even after surgical resolution of the primary lesion. The presence of inflammatory infiltrates and hemorrhagic foci likely contributed to localized myocardial irritability and delayed conduction, thereby explaining the persistent epsilon wave in the absence of ARVC. Future investigations should evaluate the prognostic value of integrating cytokine profiling and immunophenotyping in predicting post-myxoma electrophysiologic remodeling. Additionally, targeted immunomodulation, including cytokine-specific blockade, may represent a novel therapeutic approach for high-risk patients.
